# Association between different insulin resistance indices and all-cause mortality in patients with diabetic kidney disease: a prospective cohort study

**DOI:** 10.3389/fendo.2024.1427727

**Published:** 2025-01-13

**Authors:** Huan Zhu, Yinmei Chen, Dexin Ding, Hui Chen

**Affiliations:** Department of Urology, Harbin Medical University Cancer Hospital, Harbin, China

**Keywords:** diabetic kidney disease, insulin resistance, diabetes, mortality, NHANES

## Abstract

**Aim:**

Previous research has shown a strong association between insulin resistance (IR) and both the onset and advancement of diabetic kidney disease (DKD). This research focuses on examining the relationship between IR and all-cause mortality in individuals with DKD.

**Methods:**

This study utilized data obtained from the National Health and Nutrition Examination Survey (NHANES), spanning the years 2001 to 2018. Insulin resistance was assessed using reliable indicators (HOMA-IR, TyG, TyG-BMI, and METS-IR). The relationship between IR indices and survival outcomes was evaluated through weighted multivariate Cox regression, Kaplan-Meier survival analysis, and restricted cubic spline (RCS) modeling. To examine non-linear associations, the log-likelihood ratio test was employed, with piecewise regression models used to establish confidence intervals and identify threshold values. Diagnostic precision and efficacy were gauged using Receiver Operating Characteristic (ROC) curves, Area Under the Curve (AUC) evaluations, and calibration plots. Moreover, to verify the consistency of our results, stratified analyses and interaction tests were conducted across variables including age, gender, Body Mass Index (BMI), hypertension, and cardiovascular status.

**Results:**

This research involved a group of 1,588 individuals diagnosed with DKD. Over a median observation period of 74 months, 630 participants passed away. Using weighted multivariate Cox regression along with restricted cubic spline modeling, we identified non-linear associations between the four insulin resistance indices and all-cause mortality. An analysis of threshold effects pinpointed essential turning points for each IR index in this research: 1.14 for HOMA-IR, 9.18 for TyG, 207.9 for TyG-BMI, and 35.85 for METS-IR. It was noted that levels below these thresholds inversely correlated with all-cause mortality. In contrast, values above these points showed a significantly positive correlation, suggesting heightened mortality risks. The accuracy of these four IR metrics as indicators of all-cause mortality was confirmed through ROC and calibration curve analyses.

**Conclusion:**

In patients with DKD, an L-shaped association is noted between HOMA-IR and all-cause mortality, while TyG, TyG-BMI, and METS-IR exhibit U-shaped relationships. All four IR indices show good predictive performance.

## 1 Introduction

Diabetic kidney disease (DKD) represents a significant long-term complication of diabetes, primarily impacting kidney function in individuals with this condition. Approximately 20% to 40% of individuals with diabetes will develop DKD (1, 2), characterized by persistent proteinuria, declining renal function, and elevated blood pressure (3). In 2017, the United States spent an estimated $327 billion annually on managing diabetes and its related complications (4). With the rising number of individuals affected by diabetes, this cost is projected to exceed $2.1 trillion by 2030 (5). Globally, DKD not only significantly contributes to end-stage renal disease (ESRD) but also contributes to a significant number of deaths annually due to renal failure and cardiovascular complications (6–8). With the increasing incidence of diabetes, DKD has emerged as an important global health burden. Hence, implementing more effective preventive measures along with promoting early diagnosis and intervention is crucial for improving patient quality of life and alleviating the strain on public health systems.

Insulin resistance (IR) is a pathological state characterized by the impaired cellular response to insulin, a hormone that regulates glucose and lipid metabolism (9). This condition frequently coexists with other health issues, including obesity, elevated blood pressure, and lipid abnormalities (10). The hyperinsulinemic-euglycemic clamp has long been considered the gold standard for assessing insulin resistance (11). However, its requirement for specialized equipment, lengthy testing duration, and high costs make it impractical for routine clinical use. In contrast, newer alternative measures of IR, such as the Homeostatic Model Assessment of Insulin Resistance (HOMA-IR), the Metabolic Score for Insulin Resistance (METS-IR), the Triglyceride Glucose Index (TyG), and the Triglyceride Glucose-Body Mass Index (TyG-BMI), offer a more feasible approach (12–15). These alternatives are favored for their practicality, lower cost, and efficient resource utilization, providing a simpler method for measurement. Recent research has indicated that IR plays a role in kidney damage and disease progression through various mechanisms, including hyperfiltration, increased renal vascular resistance, and systemic hypertension (16–19). Collectively, these factors exacerbate the renal burden and may hasten the transition from mild kidney damage to complete renal failure.

While numerous studies have explored the predictive potential of IR indices in different populations (20, 21), their relationship with diabetes and DKD remains incompletely understood. Studies have indicated that IR, measured by estimated glucose disposal rate (eGDR), acts as an independent predictor of all-cause mortality in people with type 2 diabetes, with this relationship remaining significant even after adjusting for variables such as DKD (22). Another study found a negative association between TyG and TyG-BMI indices and all-cause mortality among stage 1–4 chronic kidney disease patients not undergoing renal replacement therapy (23). Further understanding of the predictive value of different IR indices in DKD patients, particularly in relation to all-cause mortality, is essential for precise risk assessment and individualized treatment.

This research employed data from NHANES to perform a prospective investigation into the association between IR indices and all-cause mortality in U.S. adults with DKD, exploring possible nonlinear associations between these variables.

## 2 Materials and methods

### 2.1 Study population

**Figure 1:**
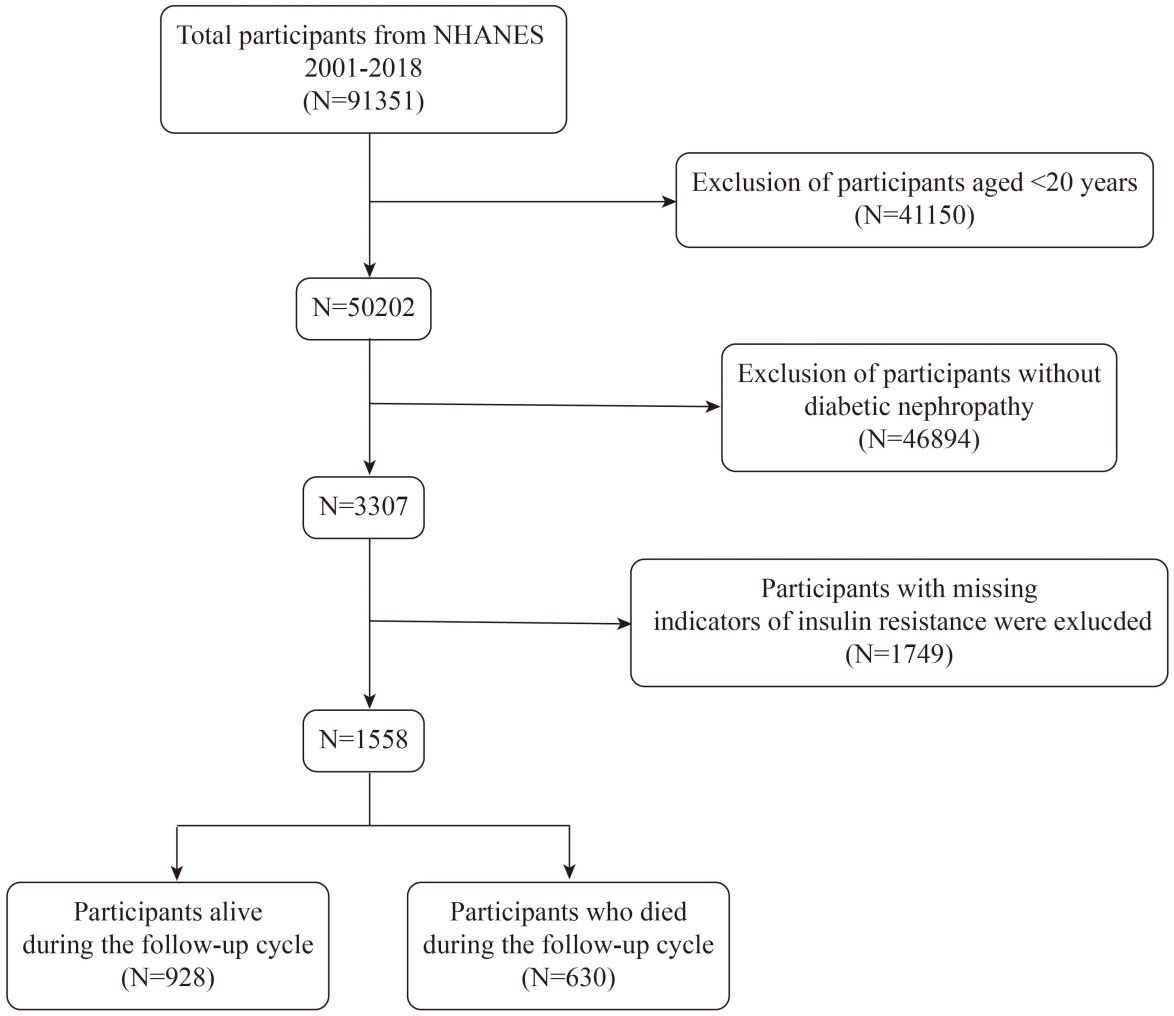
Flow chart of participants selection. NHANES, National Health and Nutrition Examination Survey.

NHANES is a comprehensive survey aimed at producing descriptive statistics that evaluate and oversee the physical health and nutrition of America’s non-institutionalized civilian populace (24). The National Death Index (NDI), maintained by the CDC, serves as a centralized database of death records. It is connected with NHANES mortality files to monitor outcomes and determine causes of death. Due to disruptions in NHANES data collection caused by the COVID-19 pandemic, data from the 2019–2020 cycle is incomplete and lacks national representativeness. Furthermore, the most recent mortality data available in the NDI only extends to 2019. Therefore, to ensure data completeness and reliability, this study includes only participants diagnosed with DKD from NHANES cycles spanning 2001 to 2018. Diabetes was identified through one or more of these criteria: clinical diagnosis, use of glucose-reducing medications, Hemoglobin A1c (HbA1c) levels ≥6.5%, Oral Glucose Tolerance Test (OGTT) results ≥200 mg/dL, or fasting plasma glucose levels ≥126 mg/dL (25). DKD was characterized by a Glomerular Filtration Rate (GFR) of less than 60 mL/min/1.73 m² or a urinary albumin-to-creatinine ratio (UACR) above 30 mg/g (26). Participants younger than 20 years and those with incomplete data on DKD or IR indices were excluded, leaving a total of 1588 participants with complete data sets for the analysis (**Figure 1**).

### 2.2 Indices for assessing IR

Our study includes four indices for assessing insulin resistance: HOMA-IR, based on fasting glucose and insulin levels, is a widely validated tool for use in both clinical and epidemiological settings (27). The TyG index provides a reliable alternative for estimating IR without direct insulin measurements (28). TyG-BMI enhances accuracy particularly in populations with obesity (29), while METS-IR is versatile and applicable across diverse populations (15). Together, these metrics offer a comprehensive evaluation of insulin resistance from multiple perspectives, improving the robustness and applicability of the methodology. These indices were derived from laboratory data using the following formulas:


$$\begin{array}{c} HOMA-IR\\ =\frac{Fasting\;Insulin\;\left(\mu U/mL\right)\times Fasting\;Glucose\;\left(mg/dL\right)}{405} \end{array}$$

$$\begin{array}{c} TyG=ln[Fasting\;Triglycerides\;\left(mg/dL\right)\\ \times Fasting\;Glucose\;\left(mg/dL\right)÷2] \end{array}$$

TyG−BMI=TyG×BMI

$$\begin{array}{c} METS-IR\\ =\frac{ln\left[2\times Fasting\;Glucose\;\left(mg/dL\right)+Fasting\;Triglycerides\;\left(mg/dL\right)\right]\times BMI}{ln\left[HDL\;Cholesterol\;\left(mg/dL\right)\right]} \end{array}$$

### 2.3 State of survival

In NHANES, all participants with adequate identifying information (such as date of birth, first and last names, gender, or Social Security Number) are eligible for mortality tracking. The primary method for determining the mortality status of eligible participants involves matching survey data with the NDI, supplemented by confirmation through death certificates from the National Center for Health Statistics (NCHS). The follow-up period begins when participants undergo their NHANES examination, with the mortality tracking data last updated on December 31, 2019.

### 2.4 Covariables

The study variables were categorized into demographic factors, disease states, and biochemical markers. Demographic variables included age, gender, race, educational level, BMI, and income-to-poverty ratio (PIR). Disease state variables encompassed hypertension and cardiovascular diseases (CVDs), with hypertension defined by systolic blood pressure ≥140 mmHg, diastolic blood pressure ≥90 mmHg, self-reported diagnosis, or the ongoing use of antihypertensive drugs (30). The determination of cardiovascular diseases included self-reported myocardial infarctions, angina, coronary artery disease, and stroke (31). Biochemical indicators included aspartate transaminase (AST), alanine transaminase (ALT), alkaline phosphatase (ALP), triglycerides, serum calcium, serum phosphorus, total cholesterol, and creatinine. These indices collectively reflect the physiological and health status of the participants, providing essential data for comprehensive analysis.

### 2.5 Statistical analysis

Participants were categorized by survival status. Weighted chi-square tests were applied to categorical variables, while weighted t-tests were used for continuous variables. Missing data were managed using random forest multiple imputation. Statistical analysis was performed in R (version 4.2.0) and EmpowerStats (version 4.2), with significance defined as p<0.05.

In order to control for potential confounding factors that could impact the analysis, we systematically constructed three distinct statistical models, each incorporating varying levels of adjustments for different covariates. Model 1 included no adjustments, allowing us to observe the raw associations between the study variables and outcomes. Model 2 introduced minimal adjustments, accounting specifically for age, gender, and ethnicity, key demographic variables known to influence health outcomes. Model 3 included comprehensive adjustments for age, gender, ethnicity, educational level, PIR, BMI, ALT, AST, ALP, serum creatinine, total cholesterol, triglycerides, serum calcium, serum phosphorus, hypertension, and cardiovascular disease status. Restricted cubic splines and log-likelihood ratio tests were utilized to explore nonlinear relationships between insulin resistance indices and survival status. Upon identifying nonlinearity, segmented regression models were utilized to determine confidence intervals and thresholds. Survival outcomes were analyzed using Kaplan-Meier methods and LogRank tests. Diagnostic performance and accuracy of the IR indices were evaluated through ROC curves, AUC, and calibration curves. In addition, stratification and interaction tests were performed for age, sex, BMI, hypertension, and cardiovascular disease.

## 3 Results

### 3.1 Baseline characteristics by survival status dichotomy

**Table 1** presents the demographic characteristics of the 1,588 participants with DKD, categorized by survival status. Among these participants, 630 passed away over a median follow-up duration of 74 months. Compared to survivors, deceased participants were more likely to be male (52.04%), older (mean age 70.27 ± 0.54 years), non-Hispanic white (72.65%), and with hypertension (86.83%). Notably, the deceased group exhibited significantly lower levels of METS-IR (*P*<0.001), TyG (*P*=0.019), and TyG-BMI (*P*<0.001) than the control group.

**Table 1:** Basic characteristics of participants by survival status dichotomy among U.S. adults.

	Total	Surviving participants	Dead participants	P-value
N	1558	928	630	
Age (years)	64.64 ± 0.44	61.21 ± 0.65	70.27 ± 0.54	<0.001
PIR	2.49 ± 0.06	2.65 ± 0.08	2.22 ± 0.07	<0.001
BMI, (%)	32.18 ± 0.25	32.86 ± 0.32	31.07 ± 0.38	<0.001
ALT (U/L)	25.81 ± 0.61	26.71 ± 0.86	24.32 ± 0.85	0.054
AST (U/L)	26.10 ± 0.51	25.53 ± 0.65	27.04 ± 0.73	0.115
ALP (IU/L)	76.22 ± 1.12	74.09 ± 1.16	79.73 ± 2.14	0.019
Calcium (mmol/L)	2.35 ± 0.01	2.35 ± 0.01	2.36 ± 0.01	0.081
Phosphorus (mmol/L)	1.20 ± 0.01	1.18 ± 0.01	1.23 ± 0.01	<0.001
Serum creatinine (mg/dL)	106.25 ± 2.8	96.44 ± 2.47	122.36 ± 5.61	<0.001
TC (mg/dL)	187.95 ± 1.74	188.90 ± 2.16	186.40 ± 2.49	0.418
TG (mg/dL)	195.48 ± 7.16	204.63 ± 10.73	180.45 ± 6.41	0.051
Gender, (%)				0.618
Male	818 (50.97%)	468 (50.31%)	350 (52.04%)	
Female	740 (49.03%)	460 (49.69%)	280 (47.96%)	
Races, (%)				<0.001
Mexican American	279 (8.90%)	191 (10.95%)	88 (5.53%)	
Other Hispanic	128 (4.78%)	95 (5.70%)	33 (3.27%)	
Non-Hispanic White	666 (64.96%)	319 (60.28%)	347 (72.65%)	
Non-Hispanic Black	352 (13.20%)	217 (13.74%)	135 (12.30%)	
Other Races	133 (8.16%)	106 (9.32%)	27 (6.25%)	
Educational levels, (%)				<0.001
< high school	612 (28.99%)	327 (23.73%)	285 (37.63%)	
High school or GED	355 (27.12%)	210 (27.28%)	145 (26.87%)	
> high school	591 (43.89%)	391 (48.99%)	200 (35.49%)	
Hypertension, (%)				0.001
No	272 (18.29%)	174 (21.40%)	98 (13.17%)	
Yes	1286 (81.71%)	754 (78.60%)	532 (86.83%)	
CVDS				<0.001
No	1072 (69.22%)	701 (74.85%)	371 (59.96%)	
Yes	486 (30.78%)	227 (25.15%)	259 (40.04%)	
HOMA_IR	9.27 ± 0.45	9.49 ± 0.54	8.92 ± 0.75	0.524
TyG	9.34 ± 0.02	9.38 ± 0.03	9.27 ± 0.04	0.019
TyG_BMI	301.61 ± 2.70	309.08 ± 3.49	289.33 ± 3.80	<0.001
METS_IR	52.16 ± 0.51	53.68 ± 0.66	49.65 ± 0.70	<0.001

Mean ± SD for continuous variables: the P value was calculated by the weighted linearregression model; (%) for categorical variables: the P value was calculated by the weighted chi-square test. PIR, the ratio of income to poverty; BMI, body mass index; ALT, alanine aminotransferase; AST, aspartate aminotransferase; ALP, Alkaline phosphatase; TC, total cholesterol; TG, triglycerides; CVDs, cardiovascular diseases; HOMA-IR, Homeostatic Model Assessment for Insulin Resistance; TyG, Triglyceride Glucose Index; TyG-BMI, Triglyceride Glucose-Body Mass Index; METS-IR, Metabolic Score for Insulin Resistance.

### 3.2 IR indices and all-cause mortality with DKD

**Figure 2:**
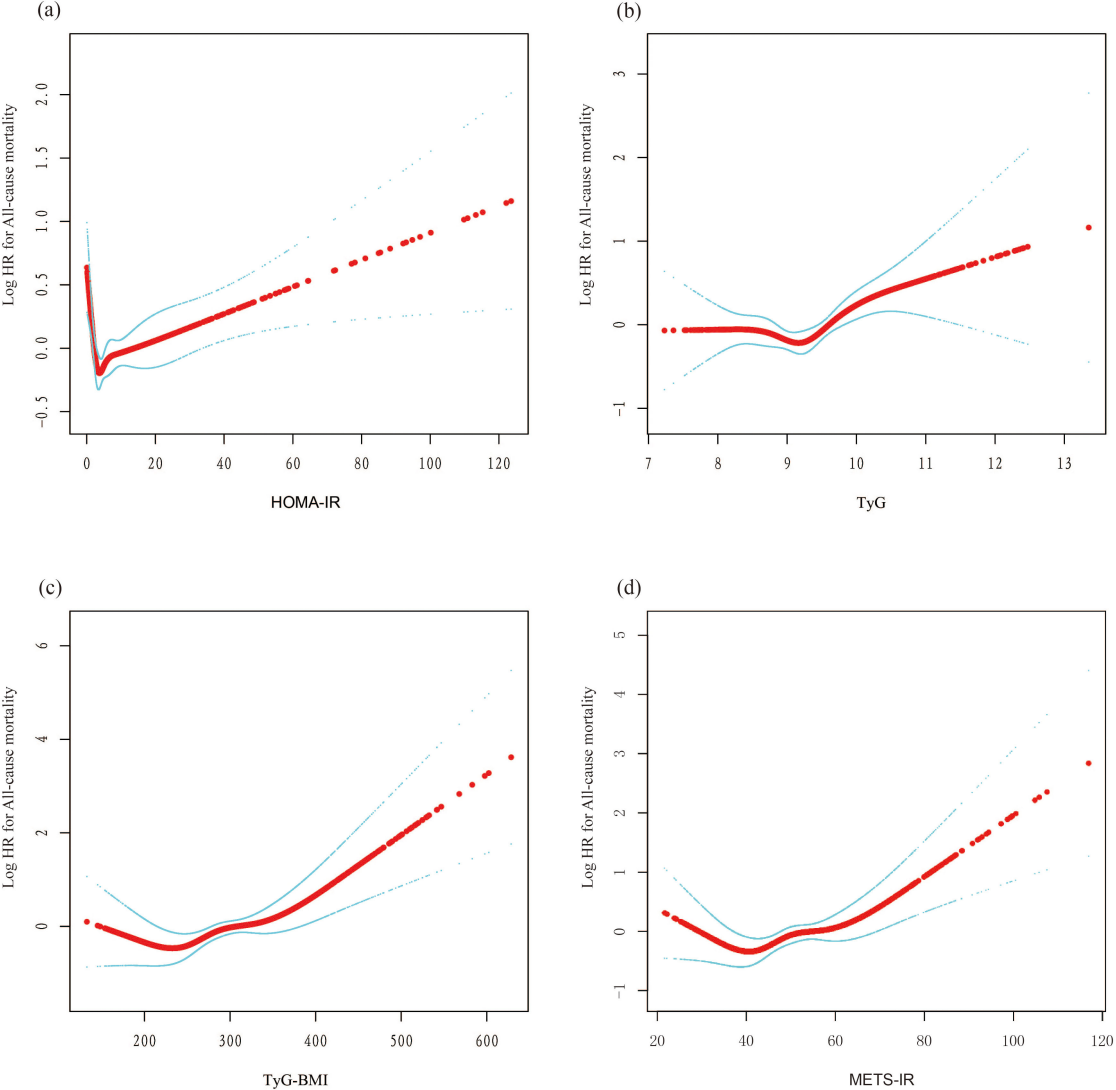
The nonlinear associations between insulin resistance and mortality. The solid red line represents the smooth curve fit between variables. Blue bands represent the 95% confidence interval from the fit. **(A)** HOMA-IR; **(B)** TyG; **(C)** TyG-BMI; **(D)** METS-IR. L-shaped association: This describes a non-linear relationship in which an increase in the independent variable leads to a rapid decrease in the dependent variable, followed by a leveling off. This pattern resembles the shape of the letter 'L' on a graph. U-shaped association: This refers to a non-linear relationship where, as the value of the independent variable changes from low to high, the dependent variable is high at both extremes (low and high values) and lower in the middle. This relationship forms a pattern resembling the letter 'U' on a graph.

**Figure 3:**
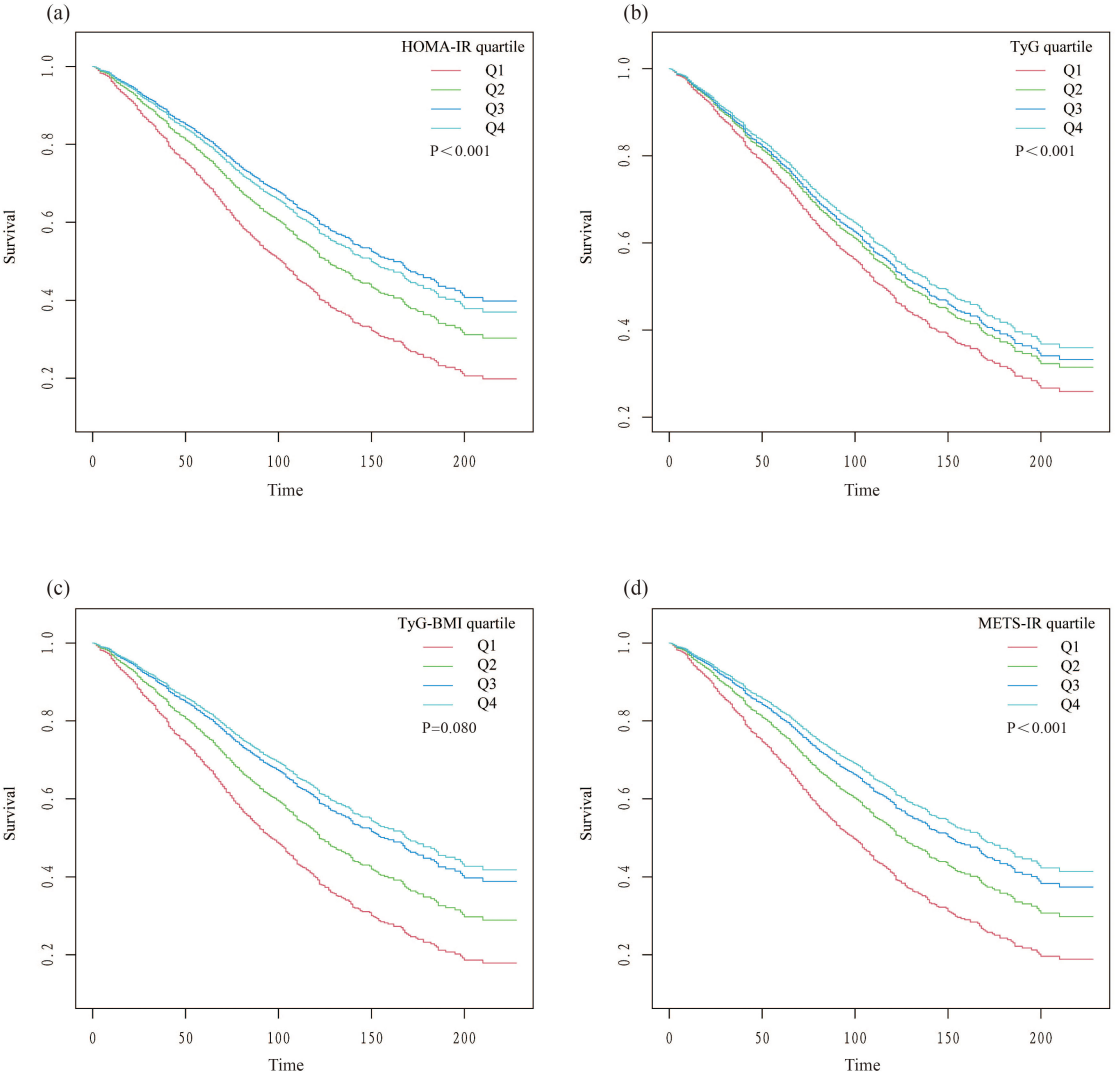
Kaplan-Meier survival curves stratified by quartiles of insulin resistance indices. **(A)** HOMA-IR; **(B)** TyG; **(C)** TyG-BMI; **(D)** METS-IR.

Findings from the multivariate Cox regression analysis revealed that, with the HOMA-IR index incorporated as a continuous variable in the fully adjusted model, each one-unit increase in the HOMA-IR index is associated with a 1% rise in all-cause mortality risk (HR 1.01, 95% CI 1.00-1.02, P=0.016) (**Table 2**). Similarly, each additional unit of the TyG index correlates with a 17% higher mortality risk (HR 1.17, 95% CI 1.01-1.36, P=0.033), and each unit augmentation in the TyG-BMI index corresponds to a 1% escalation in risk (HR 1.01, 95% CI 1.00-1.01, P=0.005). The restricted cubic spline curves illustrate an L-shaped association between HOMA-IR and mortality. At lower levels, HOMA-IR is negatively associated with mortality risk, but after a certain threshold, mortality risk increases, forming an L-shaped pattern. In contrast, the curves for TyG, TyG-BMI, and METS-IR exhibit U-shaped associations, indicating that both high and low extremes of these indices are associated with an increased mortality risk, while moderate levels are associated with a lower risk (**Figure 2**). Additionally, Kaplan-Meier survival analysis (log-rank test, P < 0.001) demonstrated significant disparities across the different HOMA-IR, TyG, and METS-IR quartiles. The survival rates were notably decreased in the lower quartiles compared to the higher quartiles (**Figure 3**).

**Table 2:** Relationship between insulin resistance and all-cause mortality in diabetic kidney disease patients.

	Model 1 HR (95%CI)	P	Model 2 HR (95%CI)	P	Model 3 HR (95%CI)	P
**HOMA-IR**	1.00 (0.99,1.01)	0.998	1.01 (1.00,1.01)	0.092	1.01 (1.00,1.02)	0.016
HOMA-IR quartile						
Quartile 1	1		1		1	
Quartile 2	0.67 (0.50,0.88)	0.005	0.70 (0.52,0.94)	0.018	0.76 (0.57,1.01)	0.600
Quartile 3	0.61 (0.46,0.82)	0.001	0.83 (0.62,1.11)	0.217	0.88 (0.64,1.20)	0.414
Quartile 4	0.63 (0.48,0.82)	0.001	0.90 (0.67,1.21)	0.502	1.07 (0.81,1.43)	0.597
**TYG**	0.85 (0.75,0.96)	0.006	1.05 (0.92,1.20)	0.494	1.17 (1.01,1.36)	0.033
TYG quartile						
Quartile 1	1		1		1	
Quartile 2	0.77 (0.58,1.02)	0.068	0.83 (0.64,1.09)	0.178	0.89 (0.68,1.15)	0.367
Quartile 3	0.70 (0.53,0.92)	0.012	0.82 (0.63,1.07)	0.146	0.90 (0.70,1.16)	0.425
Quartile 4	0.79 (0.62,1.00)	0.049	1.22 (0.95,1.59)	0.125	1.51 (1.17,1.96)	0.002
**TYG-BMI**	1.00 (0.99,1.00)	<0.001	1.00 (1.00,1.00)	0.890	1.01 (1.00,1.01)	0.005
TYG-BMI quartile						
Quartile 1	1		1		1	
Quartile 2	0.72 (0.54,0.96)	0.027	0.81 (0.61,1.06)	0.116	0.88 (0.62,1.24)	0.468
Quartile 3	0.55 (0.43,0.72)	<0.001	0.77 (0.60,0.99)	0.045	0.84 (0.53,1.34)	0.460
Quartile 4	0.57 (0.44,0.73)	<0.001	0.94 (0.72,1.21)	0.614	1.17 (0.54,2.53)	0.688
**METS-IR**	098 (0.98,0.99)	<0.001	1.00 (0.99,1.00)	0.841	1.02 (1.00,1.04)	0.061
METS-IR quartile						
Quartile 1	1		1		1	
Quartile 2	0.74 (0.56,0.96)	0.025	0.81 (0.62,1.08)	0.147	0.88 (0.64,1.22)	0.457
Quartile 3	0.60 (0.47,0.78)	<0.001	0.83 (0.64,1.67)	0.143	0.91 (0.61,1.37)	0.652
Quartile 4	0.56 (0.43,0.73)	<0.001	0.92 (0.69,1.23)	0.594	1.09 (0.62,1.92)	0.764

Model 1: No adjustments were made for covariates. Model 2: Adjustments were made for age, gender, and race. Model 3: Adjustments included age, gender, race, educational level, PIR, BMI, ALT, ALP, AST, serum calcium, serum phosphorus, total cholesterol, triglycerides, serum creatinine, hypertension, and cardiovascular disease status.

PIR, the ratio of income to poverty; BMI, body mass index; ALT, alanine aminotransferase; AST, aspartate aminotransferase; ALP, Alkaline phosphatase; TC, total cholesterol; TG, triglycerides; CVDs, cardiovascular diseases; HOMA-IR, Homeostatic Model Assessment for Insulin Resistance; TyG, Triglyceride Glucose Index; TyG-BMI, Triglyceride Glucose-Body Mass Index; METS-IR, Metabolic Score for Insulin Resistance.

Bold text is used for visual clarity to differentiate insulin resistance indices and does not indicate statistical significance or special emphasis.

**Table 3** displays the outcomes of the piecewise linear regression analysis, which identified 1.14 as the optimal threshold value for HOMA-IR (P for the log-likelihood ratio test < 0.001). Below this threshold, elevated HOMA-IR levels correlate with better survival outcomes (HR 0.11, 95% CI 0.04-0.35, P < 0.001). Conversely, above this threshold, an increase in HOMA-IR levels is correlated with an elevated risk of all-cause mortality (HR 1.01, 95% CI 1.00-1.02, P = 0.007). Similar effects were observed for the other three IR indices, with log-likelihood ratio tests showing statistical significance. The respective thresholds were as follows: TyG threshold at 9.18, TyG-BMI threshold at 207.9, and METS-IR threshold at 35.85.

**Table 3:** Analysis of threshold effects on all-cause mortality by insulin resistance indices in diabetic kidney disease.

	All-cause Mortality
HOMA-IR	
threshold point	1.14
HOMA-IR<1.14	0.11 (0.04, 0.35) 0.0001
HOMA-IR>1.14	1.01 (1.00, 1.02) 0.0068
P for log likelihood ratio test	<0.001
TyG	
threshold point	9.18
TyG<9.18	0.69 (0.48, 0.99) 0.0414
TyG>9.18	1.69 (1.24, 2.30) 0.0010
P for log likelihood ratio test	0.003
TyG-BMI	
threshold point	207.9
TyG-BMI<207.9	0.98 (0.97, 1.00) 0.0310
TyG-BMI>207.9	1.01 (1.00, 1.01) 0.0014
P for log likelihood ratio test	<0.001
METS-IR	
threshold point	35.85
METS-IR<35.85	0.92 (0.85, 0.99) 0.0201
METS-IR>35.85	1.02 (1.00, 1.05) 0.0205
P for log likelihood ratio test	<0.001

HOMA-IR, Homeostatic Model Assessment for Insulin Resistance; TyG, Triglyceride Glucose Index; TyG-BMI, Triglyceride Glucose-Body Mass Index; METS-IR, Metabolic Score for Insulin Resistance.

Data are presented as hazard ratios, 95% confidence intervals, and P-values. Adjustments were made for age, gender, race, education level, Poverty Income Ratio (PIR), Body Mass Index (BMI), alanine aminotransferase (ALT), alkaline phosphatase (ALP), aspartate aminotransferase (AST), serum calcium, serum phosphorus, total cholesterol, triglycerides, serum creatinine, hypertension, and cardiovascular disease status.

### 3.3 Predictive performance of IR on all-cause mortality in individuals with DKD

**Figure 4:**
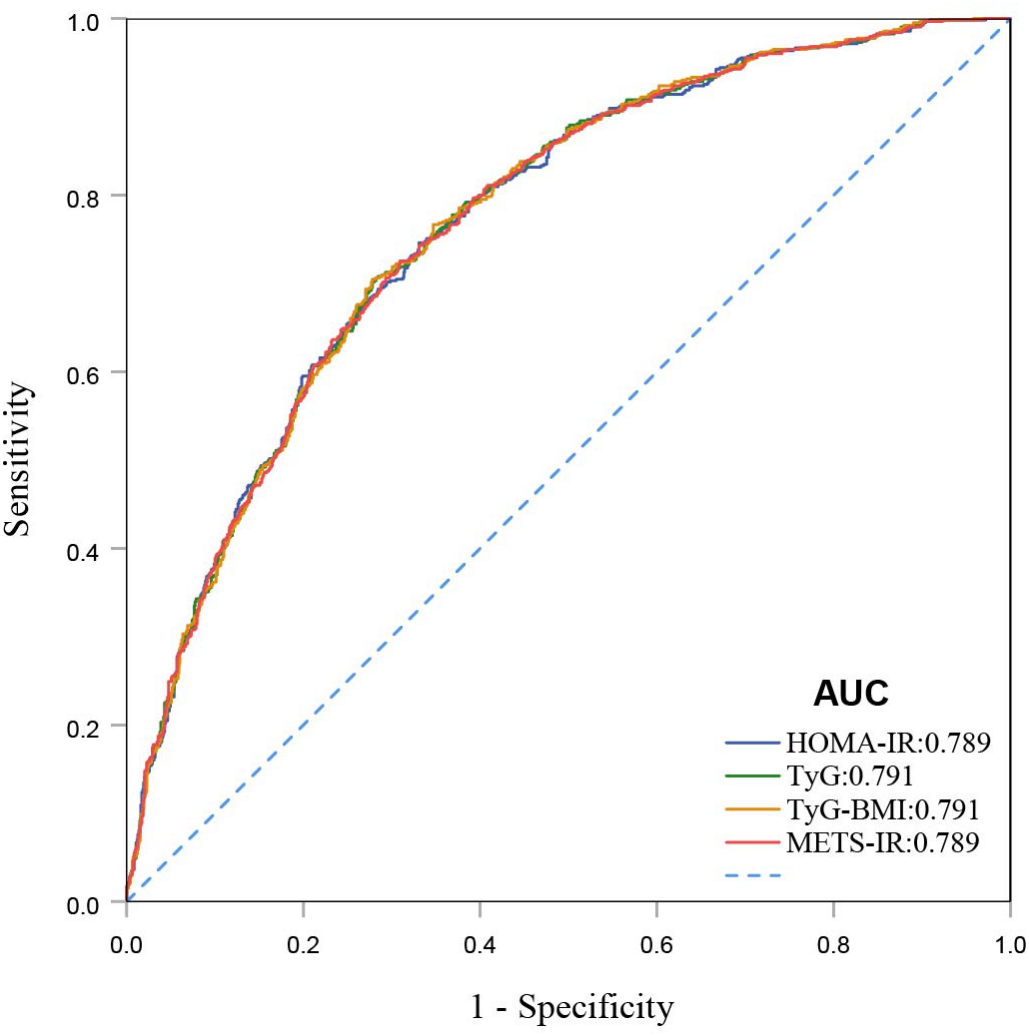
ROC curves for HOMA-IR, TyG, TyG-BMI, and METS-IR for all-cause mortality.

**Figure 5:**
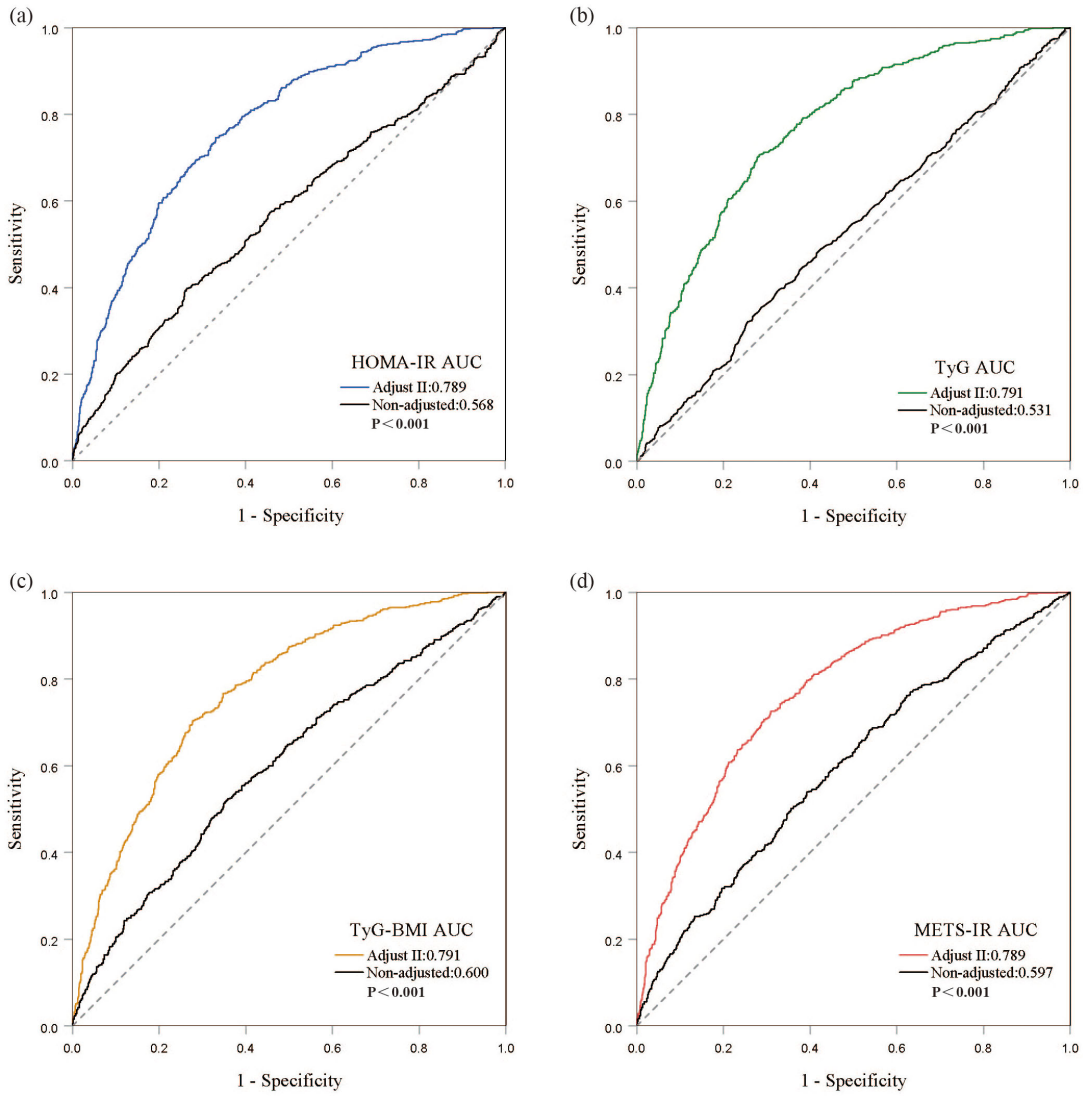
Discrimination and accuracy of four insulin resistance indices in evaluating all-cause mortality in the fully adjusted model. **(A)** HOMA-IR; **(B)** TyG; **(C)** TyG-BMI; **(D)** METS-IR.

**Figure 6:**
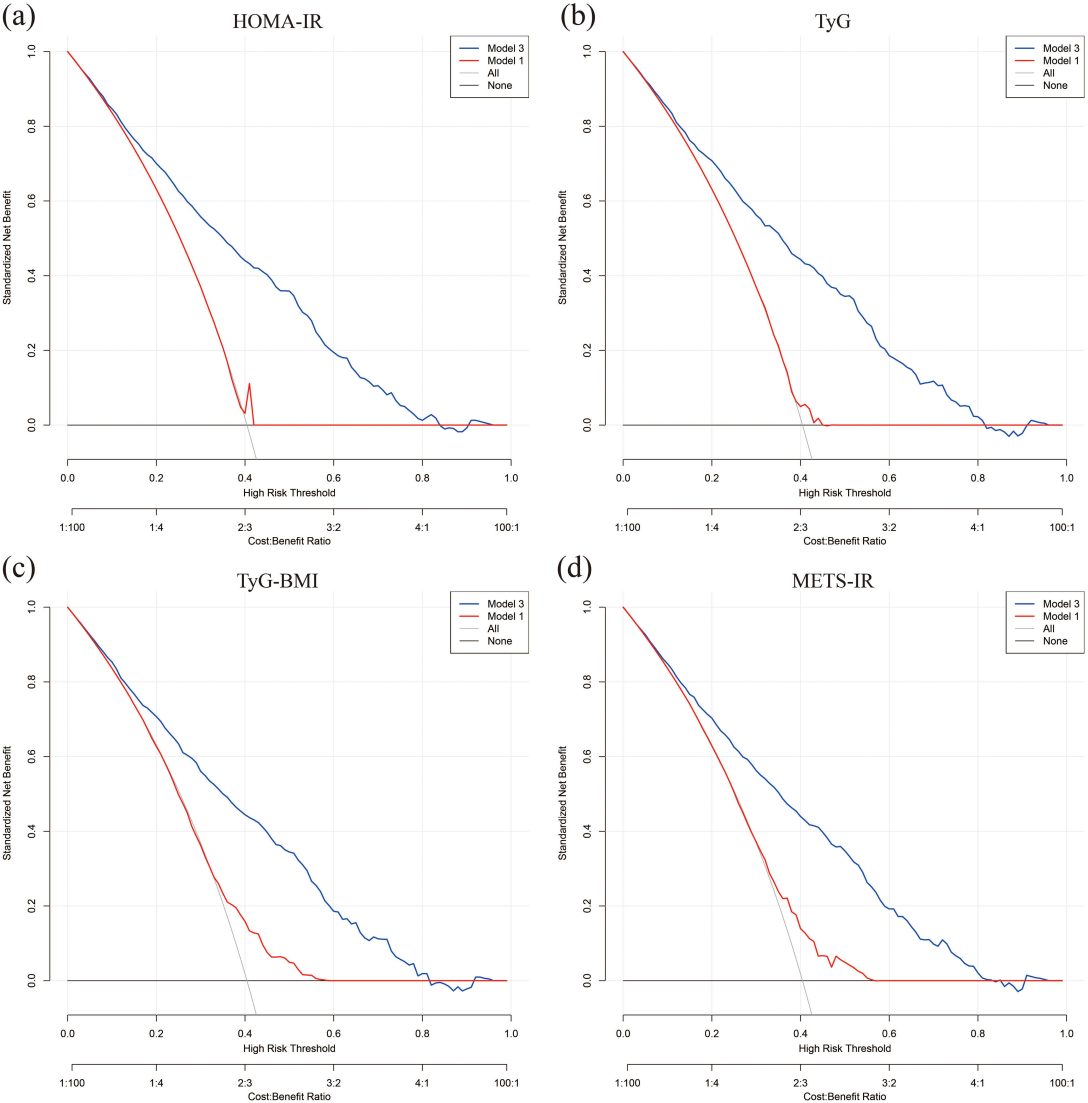
Calibration curves for model prediction accuracy. **(A)** HOMA-IR; **(B)** TyG; **(C)** TyG-BMI; **(D)** METS-IR.

ROC curves demonstrate that all four IR indices exhibit strong predictive performance for all-cause mortality, with their AUC values indicating comparable predictive abilities (**Figure 4**). When comparing fully adjusted models with non-adjusted ones, each index shows enhanced sensitivity and specificity: HOMA-IR (AUC: adjusted 0.789 vs. non-adjusted 0.568); TyG (AUC: adjusted 0.791 vs. non-adjusted 0.531); TyG-BMI (AUC: adjusted 0.791 vs. non-adjusted 0.600); and METS-IR (AUC: adjusted 0.789 vs. non-adjusted 0.597), all with P-values less than 0.001 (**Figure 5**). Calibration curve analyses corroborate these findings, suggesting that the adjusted models, which account for potential confounders, offer a more accurate estimation of all-cause mortality in patients with DKD (**Figure 6**).

### 3.4 Subgroup analyses

**Figure 7:**
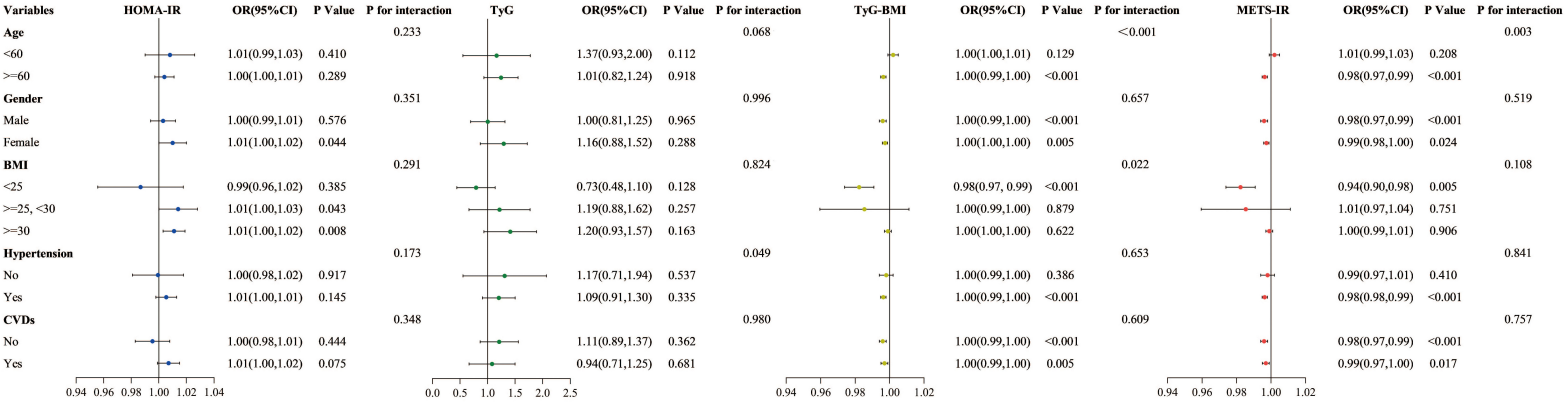
Subgroup analysis of the association between four insulin resistance indices and all-cause mortality.

The impact of factors like age, gender, BMI, hypertension, and cardiovascular disease status on outcomes was examined using subgroup analyses and interaction tests. Results revealed that the relationships of TyG-BMI and METS-IR with all-cause mortality varied significantly among age groups, showing a negative correlation specifically for participants aged 60 and above. When stratified according to BMI, TyG-BMI demonstrated a significant correlation with BMI less than 25 (HR 0.98, 95% CI 0.97-0.99, P < 0.001). With respect to other stratified variables, the analysis did not reveal any significant interaction effects (**Figure 7**).

## 4 Discussion

This study thoroughly assessed IR for its ability to predict all-cause mortality among individuals with DKD. The analysis demonstrated that all four IR indices are effective predictors of mortality outcomes. RCS and threshold effect analyses revealed a nonlinear association between these indices and mortality, indicating that both excessively low and high levels of IR pose potential mortality risks. Furthermore, significant interactions between IR and all-cause mortality across age and BMI categories were observed. In age-stratified analysis, the correlation between METS-IR and TyG-BMI levels with mortality was more pronounced among older adults. In BMI stratification, individuals with a BMI under 25 tended to experience greater survival benefits.

To our knowledge, this is the first study to assess various IR indices in relation to all-cause mortality among patients with DKD. Indeed, Previous research has shown that there is a link between IR and mortality. For example, a cohort study following participants for an average of 105 months found notable correlations between TyG and both all-cause and cardiovascular mortality among a population younger than 65 years (32). Our findings indicate that, among participants aged 60 and above, higher levels of TyG-BMI and METS-IR are unexpectedly associated with lower mortality rates. This association may reflect a phenomenon known as the “metabolic paradox”, whereby insulin resistance serves as an adaptive mechanism in older adults, potentially aiding the body in coping with external stressors (33, 34). In conditions of obesity or excess energy reserves, insulin resistance may help modulate energy management and protect against disruptions to homeostasis, thereby reducing mortality risk (35). Additionally, insulin resistance may play a protective role during aging by alleviating metabolic burden. For example, mice lacking insulin receptor substrate 1, despite exhibiting persistent insulin resistance, demonstrated reduced age-related markers of senescence and increased longevity (36). Similarly, another study found that IRKO+/− mice, which exhibit hyperinsulinemia due to insulin receptor gene knockout, show an extended lifespan (37). Thus, moderate insulin resistance may represent an evolutionarily conserved mechanism for lifespan regulation in mammals, potentially helping to reduce mortality risk.

Indeed, extensive research highlights the complex, nonlinear associations between IR levels and all-cause mortality. For instance, a retrospective study of 2,509 patients with atrial fibrillation revealed an L-shaped correlation between TyG-BMI and mortality rates (38); research on 1,126 patients with hypertension and coronary artery disease demonstrated a U-shaped correlation between HOMA-IR and the risk of mortality, indicating that excessively high and markedly low levels of insulin resistance elevate mortality risk (39); a prospective cohort study of 2,542 diabetic patients confirmed that METS-IR levels below a certain threshold significantly correlate with lower all-cause mortality risks (40). Our study similarly observed that the four IR indices were significantly negatively correlated with all-cause mortality below threshold points, while above these points, they were positively correlated, highlighting the complex nonlinear relationship between IR levels and mortality risks. This association may reflect the physiological behavior of endocrine hormones, which typically exhibit optimal effects within specific concentration ranges; deviations from these ranges can impair bodily functions (41). In patients with DKD, moderate IR may provide necessary energy reserves, aiding the body in effectively responding to acute illness or metabolic stress. Conversely, high insulin sensitivity, especially in elderly individuals, may suggest underlying chronic illness or declining metabolic function, thereby increasing mortality risk (42). Additionally, elevated insulin resistance is often associated with chronic inflammation and oxidative stress, which can exacerbate endothelial damage and kidney function deterioration, further increasing the risk of all-cause mortality (43). This nonlinear association suggests that both excessive and insufficient levels of insulin resistance may adversely impact health.

IR is crucial in the initiation and advancement of DKD. Initially, IR leads to changes in renal hemodynamics. Normally, insulin supports blood vessel dilation and stable blood flow through the activation of the phosphoinositide 3-kinase (PI3K) pathway. This pathway increases the activity of endothelial nitric oxide synthase (eNOS), facilitating the production of nitric oxide (NO) (44). In states of IR, this pathway becomes inhibited, resulting in enhanced activation of the mitogen-activated protein kinase pathway (MAPK/ERK) (16, 45), which increases endothelin-1 production, thereby elevating renal vascular resistance and decreasing renal blood flow, accelerating kidney damage (46, 47). Additionally, prolonged exposure of proximal renal tubular epithelial cells to high glucose levels leads to mitochondrial dysfunction (48). This occurs through the sodium-glucose transport protein 2 (SGLT2)-dependent pathway. This condition increases the production of inflammatory cytokines, apoptotic mediators, and oxidative stress factors, further impairing the kidney’s filtration mechanism and resulting in proteinuria and renal function decline, which are fundamental to the progression of DKD (49–51). Secondly, IR is intricately linked to chronic inflammation (52). It activates multiple inflammatory pathways, facilitating the activation and release of inflammatory cells and cytokines such as interleukin-1 (IL-1), IL-6, and TNF-α (53–55). These cytokines can directly damage renal cells, inducing endothelial dysfunction, extracellular matrix deposition, tubular cell death, and glomerulosclerosis, thereby accelerating renal function decline (56). Finally, fibrosis constitutes a critical pathological alteration in the advancement of DKD. Under conditions of IR, there is an imbalance in the actions of insulin and its related growth factors, including insulin-like growth factor 1 (IGF-1) and transforming growth factor-beta (TGF-β) (57, 58). In particular, TGF-β, a potent promoter of tissue fibrosis, stimulates the synthesis of collagen and other matrix proteins in the renal interstitium while inhibiting their degradation, leading to renal fibrosis (59). This fibrotic process is a key pathological route in the advancement of kidney disease to end-stage renal disease. The aforementioned mechanisms function in a synergistic manner to facilitate the onset and development of DKD.

Our study has several notable strengths. First, it is the only study to date that evaluates the predictive capacity of HOMA-IR, METS-IR, TyG, and TyG-BMI concerning all-cause mortality among patients with DKD. Second, the analysis utilizes data from NHANES, which implements a sophisticated sampling framework and follows rigorous quality control and standardization protocols, ensuring both data precision and national representativeness. Additionally, to enhance the reliability of the results, we adjusted for potential confounding covariates. However, it is important to recognize that this study also has certain limitations. First, the data are derived from a cross-sectional national survey, which may not fully capture the dynamic changes in IR over time. Second, due to database constraints, subtyping of the diabetic cohort was not feasible. Third, although we adjusted for a variety of conventional variables, we cannot completely exclude the impact of all potential confounders. Lastly, since the study population comprises American adults, the results may have limited applicability to populations in other regions. Therefore, future research should utilize broader samples and prospective designs to further validate these results and explore the application of IR management strategies in improving outcomes for patients with DKD.

## 5 Conclusion

The findings of our study demonstrate that four indices possess strong predictive power for all-cause mortality in patients with DKD. Each IR index shows a nonlinear relationship with all-cause mortality. Specifically, HOMA-IR exhibits an “L”-shaped curve, while TyG, TyG-BMI, and METS-IR display “U”-shaped curves. Consequently, timely identification of excessively high or low levels of IR can prompt patients to more aggressively manage diabetes and its complications, thereby reducing the risk of mortality. Nonetheless, additional research is necessary to better understand how IR indices influence mortality risk in DKD patients, especially regarding the role that distinct levels of insulin resistance might play in informing individualized treatment approaches.

## Data Availability

The datasets presented in this study can be found in online repositories. The names of the repository/repositories and accession number(s) can be found below: Detailed information about this study can be found at the NHANES online website: https://www.cdc.gov/nchs/nhanes/index.htm.
